# Poster Session I - A84 SOCIOECONOMIC FACTORS AND SYMPTOM BURDEN IN CELIAC DISEASE: RESULTS OF A NATIONWIDE CANADIAN SURVEY

**DOI:** 10.1093/jcag/gwaf042.084

**Published:** 2026-02-13

**Authors:** J A King, C McAulay, M Pinto-Sanchez, D Gidrewicz, J Turner, S Case, M Secord, D Duerksen

**Affiliations:** Community Health Sciences, University of Calgary, Calgary, AB, Canada; Canadian Celiac Association, Mississauga, ON, Canada; McMaster University Faculty of Health Sciences, Hamilton, ON, Canada; Community Health Sciences, University of Calgary, Calgary, AB, Canada; University of Alberta College of Health Sciences, Edmonton, AB, Canada; Canadian Celiac Association, Mississauga, ON, Canada; Canadian Celiac Association, Mississauga, ON, Canada; Medicine, University of Manitoba Max Rady College of Medicine, Winnipeg, MB, Canada

## Abstract

**Background:**

Prior surveys of those with celiac disease (CeD) in Canada have revealed changes in the most commonly experienced symptoms, with a growing recognition of extraintestinal manifestations of disease. Accordingly, it is worthwhile to understand if and how patient factors are associated with the severity and types of symptoms as well as their resolution after diagnosis.

**Aims:**

Identify characteristics associated with greater symptoms among Canadians living with CeD.

**Methods:**

An online survey was conducted in 2022, with information collected on domains such as demographics, symptoms, diagnosis, and management of the gluten-free diet (GFD). Cumulative link models were fit to estimate the association between respondent factors (gender, race/ethnicity, urban/rural status, income, education) with the number of symptoms experienced prior to diagnosis across three categories: gastrointestinal, extraintestinal, and psychological. Models were also fit for the likelihood of recovering from the three most common symptoms per category after initiation of the GFD. Models were adjusted for age, time since diagnosis, and comorbidities.

**Results:**

Among 7491 respondents, two-thirds (66%) reported being diagnosed by biopsy, with the remainder diagnosed by serology only or symptoms. Across all three symptom categories, there were increased odds of having more symptoms among females (F) compared to males (*p* < 0.001), whites compared to racialized persons (*p* < 0.05), rural settings (*p* < 0.001), lower income brackets (*p* < 0.001), and less than high school education compared to other education groups (*p* < 0.05). Males were more likely to recover from bloating (*p* < 0.001) and abdominal pain (*p*=0.040) than females, and an increasing likelihood of recovery for bloating, abdominal pain, and gas was associated with higher levels of income. Males were more likely than females to recover from weakness/tiredness (*p* = 0.002) and iron deficiency (*p*=0.002) than females as well as those living in urban areas or cities compared to those in rural areas (*p*<0.001). Similar trends were observed across income; there were no differences in likelihood of recovering from headaches/migraines between any respondent factor. Recovering from brain fog was higher in more urban areas (*p*=0.013); this increase was also observed as income levels increased (*p*<0.001). Males were more likely than females to recover from anxiety (*p*=0.009).

**Conclusions:**

There is notable variation in the number of symptoms experienced across demographic and socioeconomic characteristics. Additionally, these patient-level factors may be associated with greater difficulties in symptom recovery; further efforts should focus on providing support for those at increased risk of symptom-related burden such as low-income individuals.

**Funding Agencies:**

Celiac Canada

